# Exploring novel key regulators in breast cancer network

**DOI:** 10.1371/journal.pone.0198525

**Published:** 2018-06-21

**Authors:** Shahnawaz Ali, Md. Zubbair Malik, Soibam Shyamchand Singh, Keilash Chirom, Romana Ishrat, R. K. Brojen Singh

**Affiliations:** 1 Centre for Interdisciplinary Research in Basic Sciences, Jamia Millia Islamia, New Delhi-110025, India; 2 School of Computational & Integrative Sciences, Jawaharlal Nehru University, New Delhi-110067, India; University of South Alabama Mitchell Cancer Institute, UNITED STATES

## Abstract

The breast cancer network constructed from 70 experimentally verified genes is found to follow hierarchical scale free nature with heterogeneous modular organization and diverge leading hubs. The topological parameters (degree distributions, clustering co-efficient, connectivity and centralities) of this network obey fractal rules indicating absence of centrality lethality rule, and efficient communication among the components. From the network theoretical approach, we identified few *key regulators* out of large number of leading hubs, which are deeply rooted from top to down of the network, serve as backbone of the network, and possible target genes. However, p53, which is one of these *key regulators*, is found to be in low rank and keep itself at low profile but directly cross-talks with important genes BRCA2 and BRCA3. The popularity of these hubs gets changed in unpredictable way at various levels of organization thus showing disassortive nature. The local community paradigm approach in this network shows strong correlation of nodes in majority of modules/sub-modules (fast communication among nodes) and weak correlation of nodes only in few modules/sub-modules (slow communication among nodes) at various levels of network organization.

## Introduction

Breast cancer is the most common cancer in women worldwide [1], and it has the ability to get inherited which can be seen as a threat killer in an aggregated families. This inheritance is driven by some rare and common variant combinations, noted to be BRCA1, BRCA2, P53, PALB2, CHEK2 and ATM which confers high lifetime risk of the disease, while common variants at more than seventy loci identified through large-scale replication studies [2]. Till now, eradication of this disease is only through tumor surgery and chemotherapy. While surgery being the only curative approach for localized tumor (benign) of this disease revealing a high risk of advanced metastasis (malignant stage of tumor) [3]. Because of these limitations, new methods have been trying to develop with regard to primary and secondary prevention strategies of this disease. On the other hand, the bilateral mastectomy technique is only limited to women harboring BRCA1/2 germline pathologic mutations or the woman with personal history of disease [4, 5]. Based on the harmonal dependency of the breast carcinoma (the early phases), many chemoprevention techniques (primary prevention), such as, the use of selective estrogen receptor modulators (SERMs), anti-estrogen drugs (e.g. aromatase inhibitors) and micronutrients (e.g. vitamins) have been tested for anticancer activity taking the rare and common variant genes as target [2].

The ongoing paradigm of *systemics* based approaches to understand and predict mechanisms in biological phenomena is to investigate complex interaction in these biological networks and how various fundamental functions are exhibited out of the organization among the components in them. The large scale accurate omics data, high throughput gene expression data and the developed efficient integrative techniques have increasingly been used to map gene association with the specific function (biological)/disease [6]. Since mutation plays an important role (especially in cancer) in driving the associated key gene(s) to cause ultimately defective protein(s) translation which in turn perturb normal cell functioning (driving to cancer phase), these gene(s) has (have) been used as target gene(s) [7]. In breast cancer network, it has been reported the involvement of more than six thousand genes (eight thousand two hundred forty proteins) in five thousand seven hundred thirty two biological processes and one thousand nine hundred thirty molecular functions [8], and therefore it is a cumbersome task to understand the complicated organization in the network [9]. The emergent modular nature in the presently studied networks including protein-protein interaction (PPI) and metabolic networks allows us to apply *bottom-up* approach techniques to analyze the components (sub-networks/modules/communities) and their organization at various sub-levels. These network modules help us to figure out the underlying principle of progression of this disease side by side and emanate the idea of searching key target genes in the well organized breast cancer network [10]. Since modules behave as the building blocks of the higher level of functional organization of a network, they can recursively be divided in to sub-modules which become the potential source of information on the specific domain of activity [11].

Network theory approach has been proposed to be an important in understanding topological properties and the dynamics of complex systems, which can correlate to various system’s functional modules [12]. Most of the existing natural and artificial networks can fall in one of the network types namely, scale-free, small world, random and hierarchical network [13, 14]. Among them, hierarchical network is of special interest because of its important structural properties in which the appeared modules/communities with sparsely distributed hubs regulate the network [13, 14], and its self-organized working principle [13]. The presence of modules/communities in this network type is of particular interest because they may correspond to independent functional components in the network obeying their own laws [13], and exhibiting nonlinear activities [12]. The sparsely distributed hubs have an affinity to regulate and stabilize the network, within the constituting modules, but it is dubious that they are central controllers [15]. We aimed at studying breast cancer network that describe alteration (up or down regulation) in genes/pathways, which could contribute to the pathogenesis of this cancer and associated target key genes. Since the involvement of proteins in all biochemical processes is an established fact, an extensive analysis of breast cancer network constructed from protein-protein interaction network has the potential to facilitate the identification of genes affected during the disease process. Therefore, we focus our study on breast cancer network constructed from experimentally identified breast cancer genes and their interaction to explore possible important key regulatory genes. We also aim at to understand topological properties of the network from which we try to predict important key regulators among which some are of fundamental importance, their activities and regulating mechanism. We further study the complex organization in this cancer network and comment why is it difficult to crack this cancer network.

## Materials and methods

### Acquisition of breast cancer data

**Fig 1:**
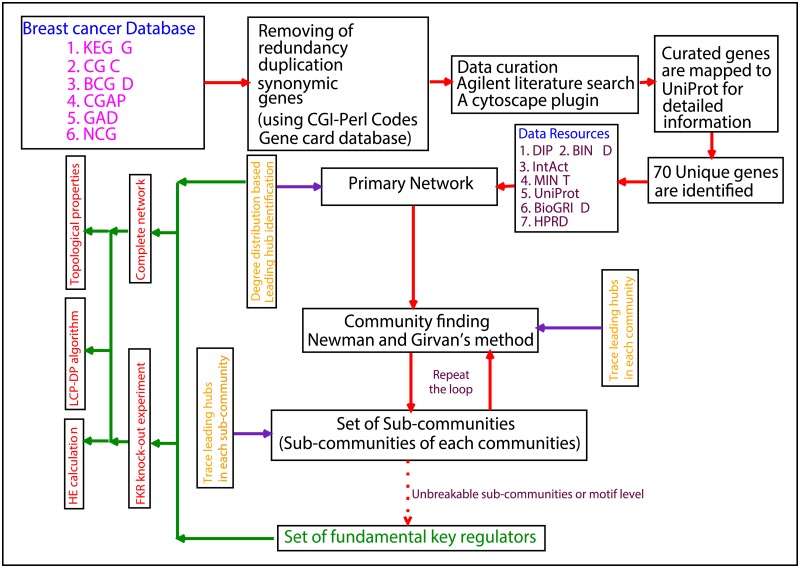
Work flow of breast cancer network construction from big data resources and method of finding *key regulators* from the constructed network with their analysis.

We have integrated six highly cited resources for cancer in order to obtain a comprehensive list of breast cancer genes. The different resources focus on different aspects of cancer biology (Fig 1); *1. KEGG* (Kyoto Encyclopedia of Genes and Genomes): it assimilates the current knowledge of molecular interaction networks, *2. CGC* (Cancer Gene Census): it defines list of those genes whose mutations are implicated in cancers, *3. BCGD* (Breast Cancer Gene Database): it accumulates the molecular genetic data related to genes involved in breast Cancer. *4. CGAP* (Cancer Genome Anatomy Project): It lists gene expression profiles inside cancer cells. *5. GAD* (Genetic Association Database): it contains genetic association studies related to cancer, that are described in literature. *6. NCG* (Network of Cancer Genes): it contains data on gene mutations. From the above assimilated repositories we have got 2050 genes, out of which 1332 were found to be unique. Again from these only 70 genes passed the test of our criterion of experimental verification. The protocol we followed for this process is a simple work flow stared with the mining of the list of genes (associated with breast cancer) from all of the six defined storehouses. These lists were subjected to CGI-Perl codes (developed locally) for the removal of duplication of both in terms of redundancy of names and use of synonymic (multiple names for the same gene) gene names. The method of removal involves pattern matching and searching globally in Gene card (http://www.genecards.com) database. This method provided the information of unique 1332 genes in *csv* format with their synonymic names. Now, this list of genes is further put into manual curation followed by the Agilent literature search, a plugin of cytoscape to get the relevant literary background on each gene. Finally, from the whole process we could able to arrived at the list of 70 genes out of 1332 unique genes. In order to construct primary network of expressed proteins we mapped these genes to UniProt (January, 2016) and got UniProt-ID, names and other functional information associated with them (70 genes).

### Construction of protein-protein interaction network

We followed one gene one protein concept to build the primary *PPI* (Protein-Protein Interaction) network of breast cancer regulatory genes (Fig 1). The network was constructed using APID2NET plug-in implemented in cytoscape version 2.8.3, which was used to retrieve all the possible information from seven main resources namely the DIP (Database of Interacting Proteins), BIND (Biomolecular Interaction Network Database), IntAct, MINT (Molecular Interactions Database), UniProt, BioGRID (The General Repository of Interaction Datasets) and HPRD (Human Protein Reference Database) [16]. The integrative and analytical effort done in APID provided an efficient open access repository where all the curated as well as experimentally verified PPIs are amalgamated into an exclusive web application. This gave us a swift exploration of interaction networks (i.e Graph denoted by G) as it includes certain parameters that weight the reliability of given interaction (i.e edge denoted by E), and also qualifying the function of any given protein with their interacting partners (i.e node denoted by V). The APID2NET parameters include (i) ‘interspecies proteins’, that filters species-specific interactions (ii) ‘hypothetical proteins’, for filtering hypothetical proteins from the interaction; (iii) ‘conexion level’, that defines the degree of the network neighborhood; and (iv) ‘experimental methods’, that defines the minimum number of methods (experimental) used to identify that particular link (edge/interaction). Here, we cross-checked this with five well studied candidate gene Tp53, BRCA1/2, EGFR, EP300 where InAct database when used gave thousands of interaction for them. On combining all the information finally we got a network of 1732 nodes harbouring 55444 interactions from which we only selected the first neighbors of selected 70 genes (discarding self-loops and isolated nodes) ending up with network of 1476 nodes defining 22314 connection between them.

### Characterization of topological properties of networks

The structural properties of complex networks can be characterized by the behaviors of the following topological parameters.

#### Degree distribution

The total number of links a node has provided by the surrounding nodes in the network is termed as the degree *k* of the node in the network. If a graph of a network is defined by *G* = (*V*, *E*), where *V* and *E* represent the sets of nodes, *V* = {*n*} and the edges between in pair of nodes *E* = {*e*_*ij*_; *i*, *j*, *i* ≠ *j*}. Then probability of degree distribution (*P*(*k*)) of the network is given by,

$$\begin{array}{c} P\left(k\right)=\frac{n_{k}}{N} \end{array}$$ (1)

where *n*_*k*_ and *N* are the number of nodes with degree *k* and size of the network respectively. For random and small-world networks, *P*(*k*) follows poisson distribution [17], whereas, for scale free network, it obeys power law *P*(*k*) ∼ *k*^−*γ*^ [17, 18] and for hierarchical networks the value of *γ* becomes close to *γ* ∼ 2.26 (mean-field value) which indicates the importance of modules with hubs in the network [13, 19].

#### Clustering co-efficient

Clustering co-efficient of a network characterize how strongly a node(s) neighborhood(s) are connected internally. It is defined as the ratio of the number of triangular motifs a node has with its nearest neighbor to the maximum possible number of such motifs. For an undirected network, clustering co-efficient of ith node can be obtained by,

$$\begin{array}{c} C\left(k_{i}\right)=\frac{2e_{i}}{k_{i}\left(k_{i}-1\right)} \end{array}$$ (2)

where, *e*_*i*_ is the number of connected pairs of nearest-neighbor of ith node, and *k*_*i*_ is the degree of the repective node. For directed network, there are in-degree and out-degree clustering co-efficients. For scale free networks *C*(*k*) ∼ *constant*, whereas, for hierarchical network it follows a power law, *C*(*k*) ∼ *k*^−*α*^, with *α* ∼ 1 [13, 19, 20].

#### Neighborhood connectivity

The average connectivity of nearest neighbors of a node in a network represents the neighborhood connectivity of the node in the network [21] which is given by,

$$\begin{array}{c} C_{n}\left(k\right)=\sum_{q}qP\left(q|k\right) \end{array}$$ (3)

where, *P*(*q*|*k*) is conditional probability that a link belonging to a node with connectivity *k* points to a node with connectivity *q*. For scale free network, *C*_*n*_(*k*) ∼ *constant*, whereas for hierarchical network, it follows power law in *k*, *C*_*n*_(*k*) ∼ *k*^−*β*^ with *β* ∼ 0.5 [22]. Further, negative and positive signs in *β* could be an indicator of disassortivity and assortivity respectively in the network topology [23].

#### Betweenness centrality

Betweenness centrality of a node in a network characterizes the ability to monitor to extract benefits from information flows in the network [24], and extent to which the node has control over the other nodes in the network through signal processing [25, 26]. If *d*_*ij*_(*v*) indicates the number of geodesic paths from node *i* to node *j* passing through node *v*, and *d*_*ij*_ represents number of geodesic paths from node *i* to *j*, then betweenness centrality (*C*_*b*_(*v*)) of a node *v* can be obtained by,

$$\begin{array}{c} C_{b}\left(v\right)=\sum_{i,j;i\neq j\neq k}\frac{d_{ij}\left(v\right)}{d_{ij}} \end{array}$$ (4)

If *M* denotes the number of node pairs excluding *v*, then normalized betweenness centrality is given by, $C_{B}\left(v\right)=\frac{1}{M}C_{b}\left(v\right)$.

#### Closeness centrality

Closeness centrality (*C*_*C*_) represents how fast information is spread from a node to other nodes reachable from it in the network [27]. *C*_*C*_ of a node *i* is defined as the reciprocal of the mean geodesic distance between the node and all other nodes connected to it in the network, and is given by,

$$\begin{array}{c} C_{C}\left(k\right)=\frac{n}{\sum_{j}d_{ij}} \end{array}$$ (5)

where, *d*_*ij*_ is geodesic path length between nodes *i* and *j*, and *n* is the total number of nodes in the network connected to node *i*.

#### Eigenvector centrality

Eigenvector centrality of a node *i* (*C*_*E*_(*i*)) in a network is proportional to the sum of *i*′s neighbor centralities [28], and it is estimated by,

$$\begin{array}{c} C_{E}\left(i\right)=\frac{1}{\lambda }\sum_{j=nn(i)}v_{j} \end{array}$$ (6)

where, *nn*(*i*) indicates nearest neighbors of node *i* in the network. λ and *v*_*i*_ are eigenvalue and eigenvector of eigen-value equation, *Av*_*i*_ = λ*v*_*i*_, where, *A* is the adjacency matrix of the network. The principal eigenvector of *A*, which corresponds to maximum positive eigenvalue λ_*max*_, represents eigenvector centrality score [29]. Since node’s eigenvector centrality function smoothly varies over the network and depends on its neighbors, node with high eigenvector centrality is embedded in the locality of nodes of high eigenvector centralities, and chance of having isolated nodes in and around the locality is very low [28]. Hence, eigenvector centrality can be used as an indicator of node’s spreading power in the network.

### Knock out experiment

To access the change of organization within the network in the absence of most influencing nodes i.e the elimination of formed Rich-clubs or leading hubs (breaking monopoly) in the network. We successively removed first five most influencing nodes from the constructed complete network, and calculated the topological properties of the modified/reorganized network to characterize regulating capabilities of the hubs by measuring the degree of structural change due to their absence. We further repeated the knock out experiment by systematic removal of 10, 20, 30, 40, 50 and 100 first leading hubs respectively to understand the role of leading hubs in the network. Every time we calculate the topological properties using Network analyser, a plug-in in cytoscape version 3.3.2, while for eigen value calculation we used CytoNCA another plug-in in cytoscape for topological properties calculation. The result from this plug-in was also helpfull in cross checking Network Analyzer.

### Community detection/finding: Leading eigen-vector method

To detect and characterize the modular nature and their properties in the hierarchical network is important in defining the predicting about the behaviour of network at various levels of hierarchy and also accessing the organizing principle of the network in study. There are many methods to detect communities, of them leading eigen vector method (LEV) gave the promising reliablity (in our case) as it calculates the eigenvalue for each link, giving importance to links not nodes. Therefore, with this believe we used LEV detection method in **R** from package ‘*igraph*’. We used this technique to detect modules from complete network, sub-modules from modules at each level of organization, and so on untill we get only motifs (i.e. 3 nodes and 3 edges). In the whole process we sticked to the criterion of identifying any sub-module as community by the presence of at least one motif (defined by *G*(3, 3).

### Estimation of network compactness: LCP-DP approach

The LCP-decomposition-plot (LCP-DP) provides one way of characterization of various topological properties of a network in two-dimensional space of common neighbors (CN) index of interacting nodes and local community links (LCL) of each pair of interacting nodes in the network. It constitutes information of number, size, and compactness of modules in a network, which can be used as an indicator of self-organization in the network [30]. Mathematically, the CN index between two nodes *x* and *y* can be obtained from the measure of overlapping between their sets of first-node-neighbors *S*(*x*) and *S*(*y*) given by, *CN* = *S*(*x*) ∩ *S*(*y*). The interaction of the two nodes could possibly take place if there is significant amount of overlapping between the sets *S*(*x*) and *S*(*y*) (large value of CN). The increase in CN could be an indication of increase in compactness in the network, which could provide faster information processing in the network. Further, the LCL between the two nodes *x* and *y*, whose upper bound is defined by, $\max\left(LCL\right)=\frac{1}{2}CN\left(CN-1\right)$, is the number of internal links in local-community (LC). The two nodes are likely to be linked together if CN of these two nodes are members of LC [30]. LCP-DP generally found to have a linear dependence between CN and $\sqrt{LCL}$. The LCP correlation (LCP-corr) is the Pearson correlation co-efficient of CN and LCL defined by $LCP-corr=\frac{cov(CN,LCL)}{\sigma_{CN}\sigma_{LCL}}$ with *CN* > 1, where *cov*(*CN*, *LCL*) is the covariance between CN and LCL, *σ*_*CN*_ and *σ*_*LCL*_ are standard deviations of CN and LCL, respectively.

### Distribution of energy in network: Hamiltonian energy calculation

The energy used in the organization of a network at a certain level/state can be measured by using Hamiltonian energy (HE) of the network at that level/state within the formalism of Constant Potts Model [31, 32]. HE provides energy distribution not only at global level of a network but also at modular level, which is in the self-organization of the system. HE of a network or module or sub-module can be calculated by,

$$\begin{array}{c} H^{\left[c\right]}=-\sum_{c}[e_{c}-\gamma n_{c}^{2}] \end{array}$$ (7)

where *e*_*c*_ and *n*_*c*_ are number of edges and nodes in a community *‘c’* and *γ* is the resolution parameter acting as edge density threshhold. Generally, we have $\gamma \leq \frac{1}{{\left(n_{c}\right)}^{2}}$. HE calculation can be beneficial in understanding the roles of modules and hubs in network organization.

## Results and discussion

### Exploration of breast cancer key genes

Interestingly, the exploration of key genes has been in a long run paving its way to even more sophisticated list [33–37] with the availability of large sets of expression data (Chip-Seq, RNA-seq and mRNA expression) and technique to analyse them. However, both the proper method and composite list of genes is yet to be formalized with practical and front-line issues of gene identification. As for instance, wu et al., 2011 has emphasized on the importance of bistable gene switches at genomic level and predicted them using mining approach [33]. In another study done by chand et al., 2012 tried make stress on 3 genes (namely CHUK, INSR and CREBBP) using mRNA expression data from SMD (Stanford Microarray Database) and Gene interaction pattern (GIP). They suggests that these 3 genes were found to be highly interacting with the breast cancer genes like Tp53, ESR1 etc [34]. while, we provided the list of key regulators that are both itself being as backbone genes and are in close link to other reported breast cancer genes. Other important studies of novel target identification done independently by [35–37] have raised this issue in a more clear genomic perspective using Chip-Seq, Gene Ontology etc in order to provide genome scale view breast cancer. We on the other hand discussed the same issue at effective communication level (i.e. first neighbours of selected 70 genes). secondly, the present study for the first time used LCP-DP approach to exploit the compactness of communities and correlate their importance in controlling network. Thirdly, we further able to show the presence of self-organization and reorganization in case of any perturbations. In addition to this we also traced down the behaviour of hubs at different level of hierarchy for this cancer. Thus we can say that our approach is more efficient in providing the better insight in synergy with expression based approaches and if overlap this network with expression data and apply our approach then it will reveal more interesting fact about the real situation going on in the network.

### Breast cancer network follows hierarchical scale-free network

**Fig 2:**
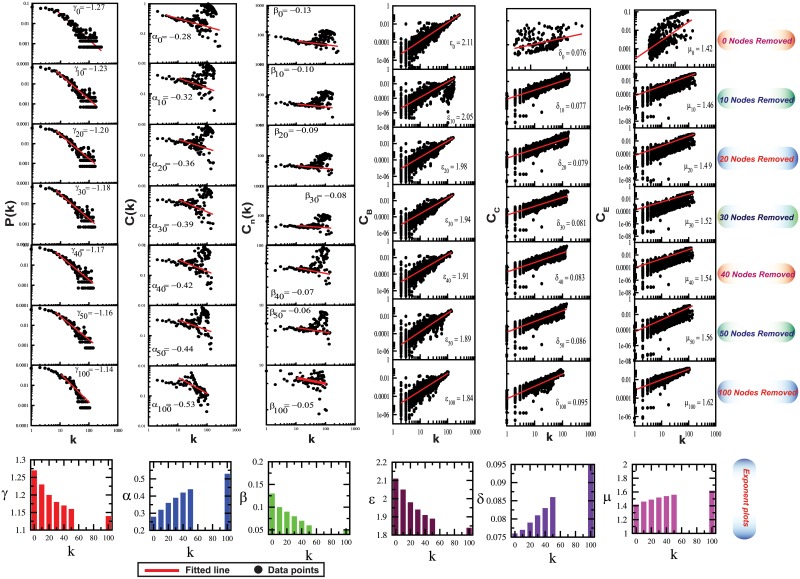
Showing the Degree distribution i.e P(k) vs. k graph, After Knock out experiment at 0, 10, 20, 30, 40, 50, 100 nodes removal and it is also fitted to the power law with exponent *γ* falling in range of Characteristic Heirarchial Networks i.e. (0 ≤ *γ* ≤ 2); Showing the Clustering Co-efficient i.e C(k) vs. k graph; After Knock out experiment at 0, 10, 20, 30, 40, 50, 100 nodes removal and it is also fitted to the power law with exponent *α* falling in range of Characteristic Heirarchial Networks i.e. (*α* ~ 1); Showing the Avg Neibourhood Connectivity i.e Cn(k) vs. k graph; After Knock out experiment at 0, 10, 20, 30, 40, 50, 100 nodes removal and it is also fitted to the power law with exponent *β* falling in range of Characteristic Heirarchial Networks i.e. (*β* ≤ 1), Showing the Betweeness Centrality, Closeness Centrality and Eigenvector Centrality i.e *C*_*b*_, *C*_*c*_ and *C*_*e*_ vs. *k* graph respectively, After Knock out experiment at 0, 10, 20, 30, 40, 50, 100 nodes removal and it is also fitted to the power law with exponent *ϵ*, *δ* and *μ* in order.

The topological parameters of the breast cancer network we constructed (Method and Fig 1) using experimentally verified seventy genes (Table 1) follow power law distributions as a function of degree. The probability of degree distributions (*P*(*k*)), clustering co-efficient (*C*(*k*)) and connectivity (*C*_*n*_(*k*)) exhibit power law or fractal nature (Fig 2 first row, three first left panels), and for complete network, it is given by,

$$\begin{array}{c} \left(\begin{array}{c} P\\ C\\ C_{n} \end{array})∼(\begin{array}{c} k^{-\gamma }\\ k^{-\alpha }\\ k^{-\beta } \end{array});\;(\begin{array}{c} \gamma_{0}\\ \alpha_{0}\\ \beta_{0} \end{array})\to (\begin{array}{c} 1.27\\ 0.28\\ 0.13 \end{array}\right) \end{array}$$ (8)

This network behavior characterized by Eq (8) indicates hierarchical scale free network [13, 19, 20, 22, 38, 39]. The power law fits on the data points of the topological parameters of the network are done and verified following a standard statistical fitting procedure proposed by Clauset et. al. [40], where, all statistical p-values for all data sets, calculated against 2500 random samplings, are found to be larger than a critical value 0.1, and goodness of fits are found to be less than and equal to 0.33. The negative value in *β*_0_ of connectivity parameter shows disassortive nature of the network, and possibility of rich-club formation among the leading hubs is unlikely [22]. However, the roles of the leading hubs are still significant in regulating the breast cancer network.

**Table 1:** List of *Key regulator* genes out of 100 leading hubs identified in breast cancer network. The colored (blue) genes are those FKRs which are already found to be very important genes in breast cancer regulation.

SL. No.	Name of FKR	Ranking among FRK	Ranking among Leading Hubs
1	EGFR	1	1
2	EP300	2	2
3	GRB2	3	3
4	BRCA1	4	8
5	CBP	4	35
6	RS3A	5	9
7	PHB	5	10
8	KU70	6	10
9	RL13	6	44
10	P53	7	26
11	RL13A	8	54
12	SF3B2	8	86
13	DHX15	8	87
14	RL17	9	55
15	ANM5	9	75
16	RL8	10	52
17	SART3	10	60
18	RL18A	10	63
19	RL10	10	64
20	IMMT	10	66
21	SYEP	10	68
22	IF4A3	10	69
23	RL28	10	70
24	RL14	10	71
25	RL19	10	72
26	DDX39	10	77
27	SYNE1	10	78
28	COPA	10	79
29	RL12	10	81
30	MBB1A	10	82
31	DICER	10	83
32	MATR3	10	85
33	RPA1	10	88
34	TCPA	10	90
35	ILF3	10	95
36	PALB2	10	96
37	TF3C1	10	98
38	RL15	10	99
39	ELAV1	10	100
40	DLX4	10	100
41	NUCL	11	53
42	RL18	13	56
43	RL7A	13	59
44	RL21	14	43

The centrality parameters, namely, betweenness (*C*_*B*_), closeness (*C*_*C*_) and eigen-vector (*C*_*E*_) centralities of the network also exhibit fractal behavior (Fig 2 first row, three right panels) given by,

$$\begin{array}{c} \left(\begin{array}{c} C_{B}\\ C_{C}\\ C_{E} \end{array})∼(\begin{array}{c} k^{\epsilon }\\ k^{\delta }\\ k^{\mu } \end{array});\;(\begin{array}{c} \epsilon_{0}\\ \delta_{0}\\ \mu_{0} \end{array})\to (\begin{array}{c} 2.11\\ 0.076\\ 1.142 \end{array}\right) \end{array}$$ (9)

The positive value of exponents of these centrality parameters, shown in Eq (9), indicate the strong regulatory role of the leading hubs in the breast cancer network [25, 29].

### Strong inter-links in breast cancer network

To understand the organization, reorganization and importance of leading hubs on the breast cancer network, the changes in the topological properties of the network are studied by removing leading hubs from the network (Fig 2). The changes in the properties of the hubs knock out networks can be captured by their exponents of stamped fractal laws, which are given by,

$$\begin{array}{c} (\begin{array}{c} \gamma_{i}\\ \alpha_{i}\\ \beta_{i} \end{array})=(\begin{array}{c} 1.27-1.14\\ 0.28-0.53\\ 0.13-0.05 \end{array});\;(\begin{array}{c} \epsilon_{i}\\ \delta_{i}\\ \mu_{i} \end{array})\to (\begin{array}{c} 2.11-1.84\\ 0.076-0.095\\ 1.142-1.62 \end{array});\;i=0,10,20,30,40,50,100 \end{array}$$ (10)

The decrease in *γ* (1.27 − 1.14 < 1 + *ln*(4)/*ln*(3) = 2.26, mean-field value of hierarchical network [19]) in Eq (10) (Fig 2 first panel of last row) favors more significant role of hubs in the hubs knocked out network [20]. This shows that removing more leading hubs from the network allows more responsibilities of the existing leading hubs in the resulting network in order to reorganize and save the network properties from break-down. The increase in *α* (Fig 2 second panel of last row) surprisingly indicates the increase in compactness in the hubs removed network in order to save the network from break down. However, decrease in network connectivity exponent *β* as increase in the number of hubs removed (Fig 2 third panel of last row) may be due to significant decrease in connections of low degree nodes associated with the removed hubs.

To understand the importance of a particular cellular function, one needs to identify molecular components (basically Genes) and the interaction among them which are related and accountable to it. One of the traditional approaches used to capture the importance of functional modules and leading hubs is to see the difference in the expression of genes through ‘Gene Knock-Out’ experiment [41]. Following this procedure, the absence of gene expressions of a small number of knock out genes from the genetic background allows one to able to access regulation of hundreds of other genes present in the network. This regulation can be revealed (theoretically) by the change in the connections and topological properties before and after knockout. Leading hubs knock out experiment allows to decrease betweenness centrality exponent *ϵ* with increase in removed hubs (Fig 2 fourth panel of last row) which reveals that the regulating roles of remaining hubs become less important [24], but the role of modules might be significantly important in the network [25]. Therefore, the controlling capability of remaining hubs become weaker, as a consequence the modules in the network might regulate the overall network activities [26]. However, the values of exponents of other centrality parameters, namely closeness and eigen-vector centralities, *δ* and *μ* increases as the number of removed hubs increases. The increase in *δ* indicates that the information processing in the network becomes faster when leading hubs are removed which means that local perturbations due to hubs removal are strong enough to cause significant change in global scenario [27]. This fast information processing might be useful in reorganizing the perturbed network to maintain the required network properties and to save the network from break down. Further, the increase in the value of exponent of eigenvector centrality *μ* reveals that each node in the network have stronger links with the rest of the network, embedded it deeper in the locality of the network, and possibility of isolation of it from the network becomes significantly less [28]. This means that the roles of modules in the perturbed network become more important to keep the network’s inherent properties and to save it from break down [28, 29]. Since the breast cancer network is hierarchical scale free network, the modules have stronger capabilities of regulating the network than the existing hubs, and removing few hubs does not cause network break down [13]. The knock out hubs experiment we performed shows no drastic change in the topological properties of the perturbed network (Eq (10)), but enhance the regulating capabilities of the modules than the hubs, which is the consequence of strong inter-links in the network.

### Absence of centrality-lethality rule in breast cancer network

**Fig 3:**
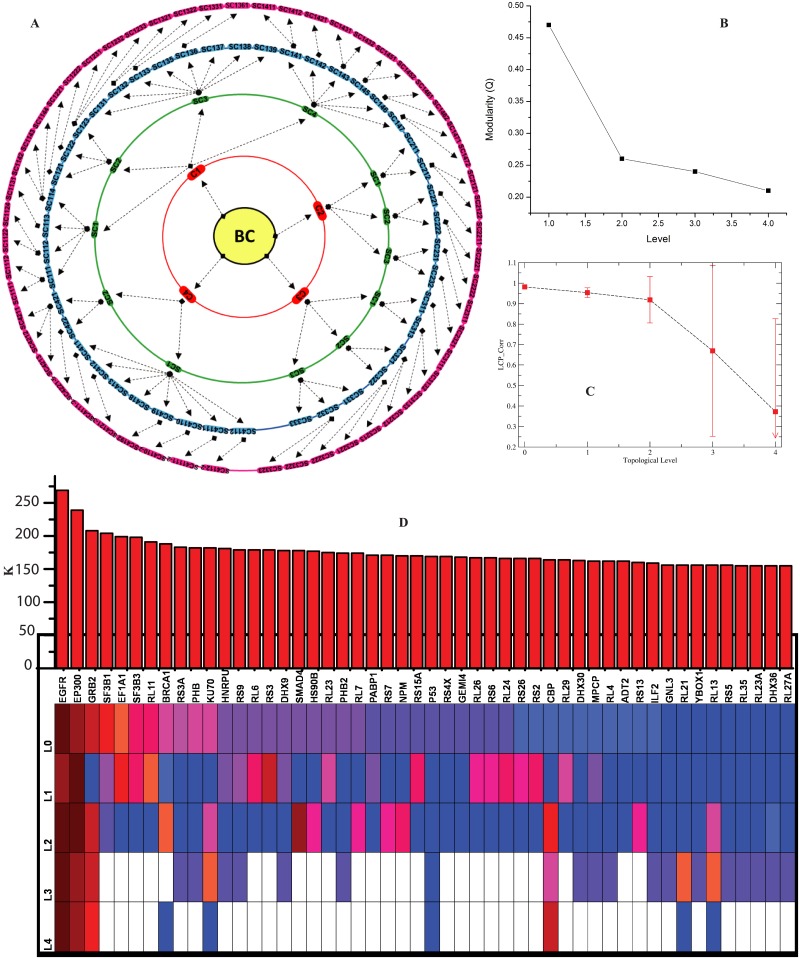
System level organization of breast cancer network. (a) Organization of modules/sub-modules at various levels (indicated by various concentric circles) and arrows show sub-modules constructed from previous modules. (b) Plots of modularity and LCP-correlation per node as a function of level of organization. (c) Popularity rankings of the first fifty leading hubs in the complete network: the plot also shows unpredictability of the these hubs at various levels of organization. Identification of *key regulators* of breast cancer network.

Since the breast cancer network falls in hierarchical scale free network, the network can be represented as a system level organization of modules/sub-modules at different levels of organization (Fig 3A) applying Girvan and Newman’s standard community finding technique [14]. Since the normalized modularity (*Q*), which is defined as the modularity per node in the network, and normalized LCP-correlation, defined as LCP-correlation per node increase as level of organization (*s*) increases (Fig 3B), the nodes in the network are tighly connected. Hence, regulation of the network by modules/sub-modules at various levels of organization dominates the regulatory roles by hubs in the network.

**Fig 4:**
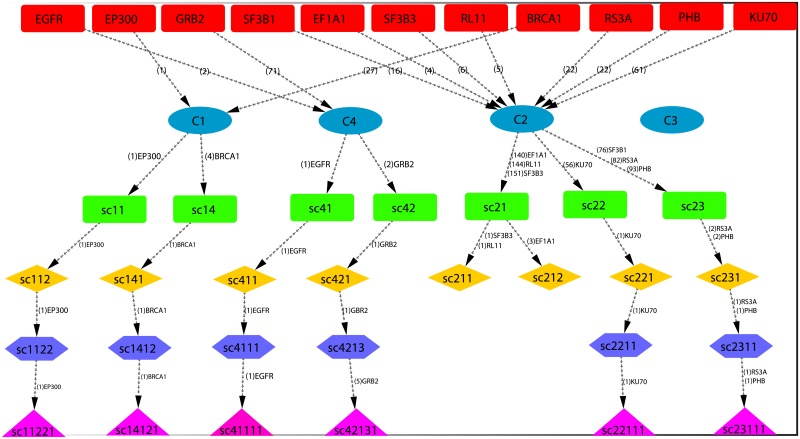
The structures of modules/sub-modules through which the first ten leading hubs passed through. The probability distribution of the seven *key regulators* as a function og level of organization.

Generally, essential genes can be identified from single gene knockout experiments one after another. However, these experiments are time consuming and limited (require set of criterion to be confirmed before application). Essential genes in biological context means genes that are highly inter-connected (or leading hubs). The accumulation of mutation in any gene results in the disfunctioning or removal of expression of the specific protein in the cell. This scenario in theory can be demonstrated by removing the corresponding hub/hubs (essential gene/genes that may have been mutated) from the network and then rechecking the topological change in the network [42]. If the removal of that/those hub/hubs cause drastic change in the network properties, such as, break-down of the network etc then the network is said to be governed by centrality-lethality rule in it [43]. However, in breast cancer network, removal of some leading hubs do not cause network break down because of its hierarchical properties (Figs 2, 3 and 4), where, the emergence of highly diverged modules at different levels of organization protects its traditional properties, and leading hubs could no longer over-regulate modular mechanisms in the network. This absence of centrality-lethality rule clearly suggests that the top leading genes, such as, EGFR, EP300, GRB2, BRCA1 etc present in the network are not the only ones that confirms the correct functioning of regulation mechanism, while they work in co-ordination manner with other comparatively less esential genes.

To understand more specifically about the competition between the regulatory roles of leading hubs and modules/sub-modules, the activities of the first fifty leading hubs are considered. The first nearly ten leading hubs show significant change in their degree *k*, but the remaining hubs show slight variations in their degrees showing nearly similar activity. If one defines the popularity of a hub at any level of organization *s* by its degree, then the popularity of a particular gene gets changed at different levels (Fig 3D). This means that a particular hub might be very popular and could have interfered various activities in the module it belongs and other modular activities in that level, whereas at other levels it may stay at low popularity without interfering various intra and inter modular activities (Fig 3D color codes indicate popularity). Hence there is no single or few hubs which control the network at various levels of organization. Therefore, removing of few leading hubs never cause network break down which is in fact absence of centrality-lethality rule in this network [43]. This absence of centrality-lethality rule or absence of central control system could be one of the most important signatures of inherent self-organization in the breast cancer network [44].

### Key regulators of breast cancer

Since the popularity of leading hubs get change with their activities and regulating mechanisms, all the leading hubs may not be key regulators for clinical and drug target genes. However, few of these leading hubs can be important, which we term as *key regulators* (*FKR*), and can be defined as the deeply rooted hub genes which can able to reach from main network to motif level (fundamental regulating unit) through various levels of organization via modules/sub-modules (Figs 3D, 4, S1 Fig). These regulators work at grassroots level with basic maintaining technologies, and are generally backbone of keeping network stability locally as well as globally. They could be key to network structural and functional integrity at various levels, and essential organizers of maintaining network stabilization whenever the network is under attack. They are generally main information propagators as well as receivers, and serves as means of cross-talks of far and near nodes in the network for possible segregation even though they are physically far away from one another. As long as the fundamental key regulators are there in the network deeply buried through large number of levels of organization, the network will have strong capability of defending any attacks, and unacceptable changes in it. Following this definition, we could able to identify eleven *key regulators* out of fifty leading hubs in the breast cancer network, which are *EGFR, EP300, GRB2, BRCA1, KU70, RS3A, PHB, P53, CBP, RL21* and *RL13* respectively. These hubs take part and regulate at any level of organization of the network starting from fundamental regulating unit i.e. motif.

The popularity of the eleven *key regulators* get changed at various levels of organization. The only first three leading hubs could able to maintain their own big popularities from top (main network) to bottom (motif level). Only one of these regulators, *p53* maintain its low popularity profile but turns out to be an important fundamental regulator in breast cancer network. Two of the eleven regulators, namely *RS3A* and *PHB*, are found to be regulating together in the same module/sub-module at various levels of organization till motif level (Fig 4). These two regulators are still maintain low profile, but important regulators.

Now the approximate regulating capabilities of these *FKR* and their activities in the networks/modules/sub-modules, where they belong to, can be estimated by defining a probability *P*_*FKR*_(*x*), which is the probability of a *FKR* to have *x*^[*s*]^ links in the network/module/sub-module it is accommodated having total *N*^[*s*]^ edges, where *s* is the level of organization index, given by,

$$\begin{array}{c} P_{FKR}\left(x^{\left[s\right]}\right)=\frac{x^{\left[s\right]}}{N^{\left[s\right]}};\;s=0,1,\ldots m;\;m=5 \end{array}$$ (11)

The calculated $P_{FKR}^{\left[s\right]}$ s for all *FKR* show their increase in values as a function of level of organization *s* (S1 Fig panels of lowest row). This indicates that capabilities of regulating of these *FKR* increase at deeper level of organization, and their activities become more prominent. Therefore these *FKR* are active workers at grassroots level, and become backbone of the network organization and stabilization. We could able to identify forty four *FKR*s out of first one hundred leading hubs in breast cancer network (Table 1).

Most of the leading hubs, except few (eleven out of fifty leading hubs), are not key regulators which are involved in fundamental regulation of cancer. This means that even though these hubs has high popularity in the complete network, they generally end up their existence after few levels of organization (Figs 3, 4, S1 Fig). We also observe that ranking of popularity of leading hubs at complete network level does not necessarily provide as an indicator of that hub of becoming a fundamental key regulator. Further, these hubs are not as important as fundamental key regulators in the long run as far as preservation of network properties is concerned. Hence, in breast cancer network fundamental key regulators could be target genes for possible diagnosis and cure of this disease.

Some of the identified *FKR* are found to be experimentally known important breast cancer regulator genes. For example, EGFR is a transmembrane tyrosine kinase receptor, which forms functionally active dimers, such as, EGFR-EGFR, EGFR-HER2, EGFR-HER3, EGFR-HER4). This dimerization induces the recruitment of a range of adaptor proteins, such as, GRB2 and then activates a cascade of intracellular signaling to alter gene transcription, which results in cancer cell proliferation, reduction of apoptosis, and metastasis [45]. Germline mutations in BRCA1/2 gene cause a significant amount of hereditary effect in breast cancer subjects. Further, most of the BRCA1 kindered breast cancers are of triple-negative phenotypes (ER negative, PR negative and HER2 negative) and harbors with TP53 somatic mutations. However, BRCA2 associated cancers are less homogeneous and often ER positive [46]. On the other hand, PHB (Prohibitin) is a negative regulator of cell proliferation and a tumor suppressor protein, and it is connected to diverse cellular functions like cell cycle control, senescence, apoptosis and regulation of mitochondrial activities. However, in different tissues/cells, it has different level of expression, its expression level is high in most of the cancers [47]. Moreover, non-homologous end-joining (NHEJ) is an important pathway for the repair of DNA double strand breaks (DSBs) in human cells. This NHEJ pathway is frequently upregulated in many cancers as a compensation for innate genomic instability, making this pathway a pious target. KU70/80 heterodimer protein, owing to the high affinity to DNA termini, serves as the central regulating factor during repair of DSBs via NHEJ pathway [48]. On the other hand, RS3A may play a role during erythropoiesis under the guidence of transcription factor DNA damage inducible transcript 3 (DDIT3) [49].

### Key regulator p53 maintain low profile

**Fig 5:**
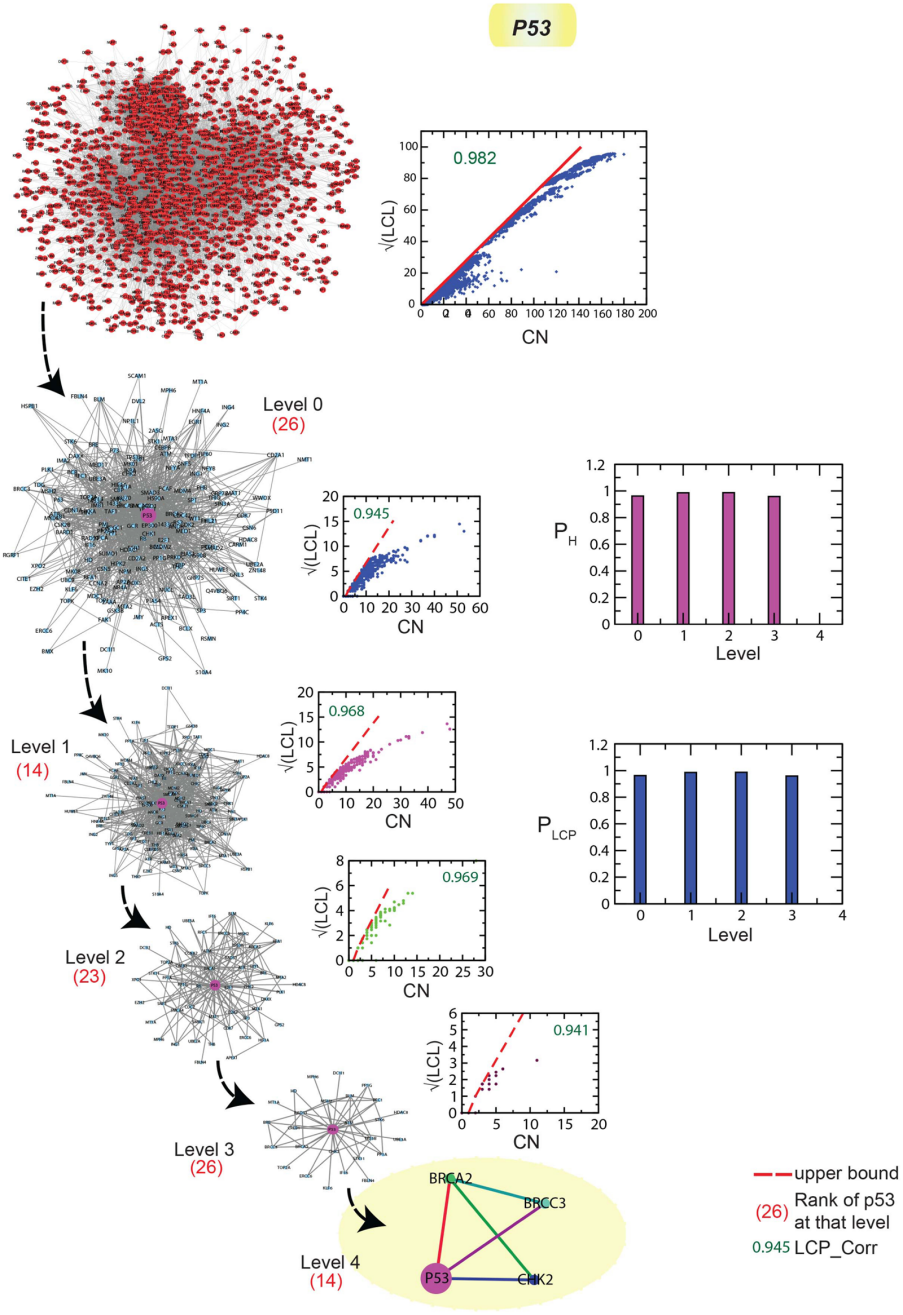
The modular path of p53 from complete network to motif with the structures of modules/sub-modules at various levels in which p53 is accommodated. (a) The plots of LCP-correlation as a function of CN for each modules/submodules (plots corresponding to each module/sub-module of the network) of p53 path. (b) The plots of *P*_*H*_ and *P*_*LCP*_ as a function of level of organization.

In breast cancer network, even though p53 is found to be *key regulator*, it keeps its profile low (its ranking of popularity is in between 14-26 in all the levels of organization) (Fig 5). However, it interacts with other important genes BRCA2, BRCA3 and CHK2 forming two triangular motifs at the lowest level of organization (*level* = 4). Since it friends with these important genes, it might directly and indirectly regulate the breast cancer mechanisms in an efficient way as compared to other key regulators. Further, the modules/sub-modules it belongs to are also tightly bound as reflected from the large values of LCP-correlation values (see Methods) which have values in the range between [0.941 − 0.969] > 0.8 [30] (Fig 5). To characterize the compactness or how strongly the nodes are interconnected in the modules/sub-modules at various levels of organization, where p53 constitutes, we define a relative LCP-correlation given by,

$$\begin{array}{c} P_{LCP}=\frac{x_{i}}{x_{N}};\;i=1,2,3,4 \end{array}$$ (12)

where, *x*_*i*_ is the value of LCP-correlation of ith level of organization and *x*_*N*_ is the LCP-correlation of the complete network. Since the *P*_*LCP*_ values calculated using Eq (12) are almost the same for various various levels (Fig 5 right lower panel), the p53 is burried in depth at each level and allowed it to regulate through its important partners.

We further calculated energy distributions in modules/sub-modules, where p53 is accommodated, using Hamiltonian function given by Eq (7) (see Methods). Defining the energy distribution per node, which is the ratio of Hamiltonian energy of a module/sub-module at a particular level ‘s’ *H*_*s*_ to the size of the corresponding module/sub-module *N*_*s*_ given by,

$$\begin{array}{c} P_{H}=\frac{H_{s}}{N_{s}};\;s=1,2,3,4 \end{array}$$ (13)

we can estimate the amount of energy distribution at each level. The calculated *P*_*H*_ using Eq (13) is found to be approximately the same for all levels of organization (Fig 5 right upper panel). This reveals that p53 modular/sub-modular organization at various levels are very much similar and close to one indicating similar and strong organization.

p53 is well studied tumor supressor gene, playing important role in signal transduction as well as regulating stress inside the cell [50, 51]. It is considered to be an important key candidate in cellular mechanisms, which is mainly responsible for cellular fate decision, either cell cycle arrest or apoptosis [52]. Further, as far as cancer diseases are concerned, p53 is found to be mutated in 50% of all human cancers [53] and 80% in basal/triple negative breast cancer [54].

### Heterogeneity in network organization: Key to resist any change

**Fig 6:**
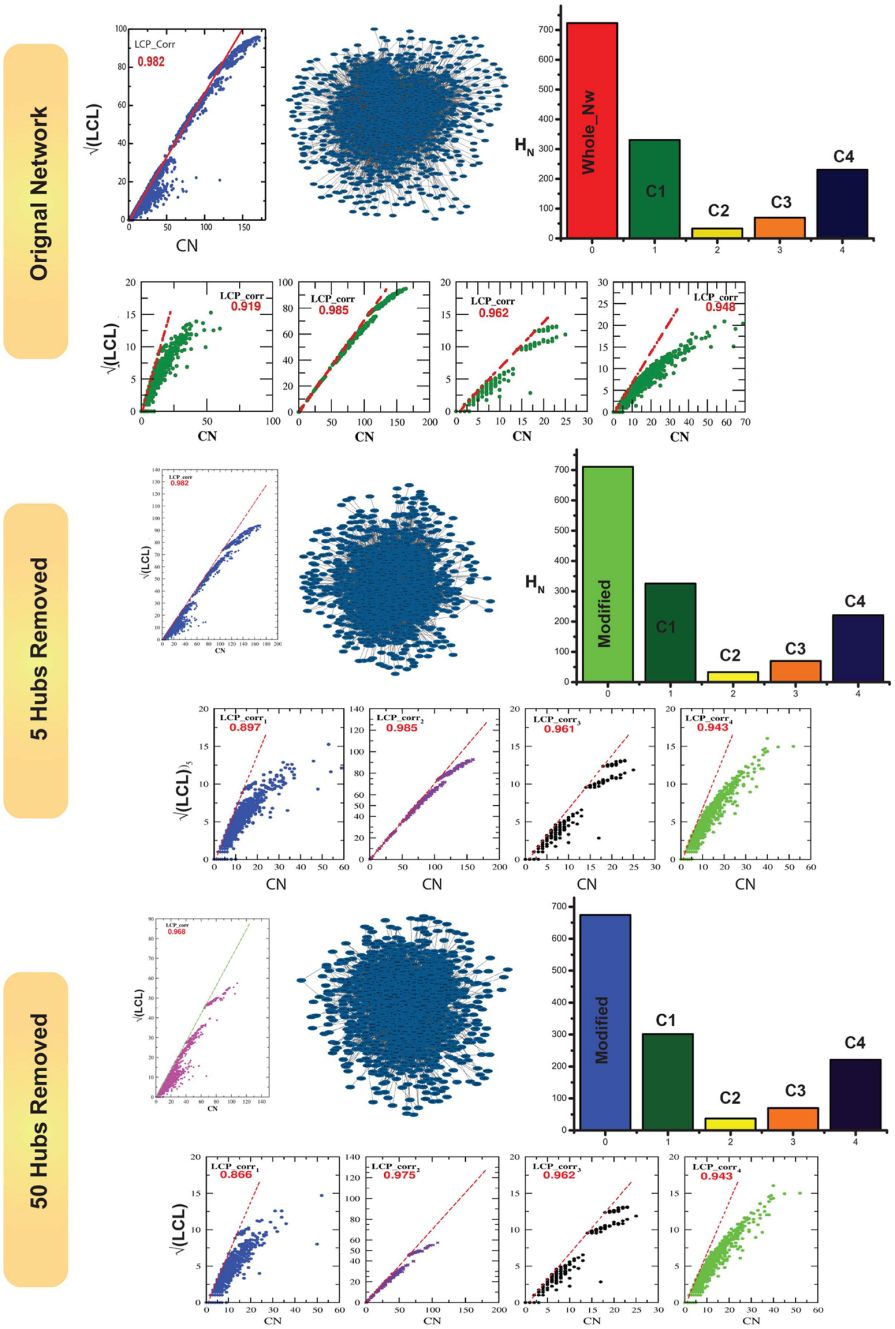
Compactness of breast cancer network: LCP-correlation calculation as a function of CN for complete and first level modules when zero, five and fifty leading hubs are removed.

The degree of compactness and nodes distribution in a network can be measured by local community paradigm: decomposition plot (LCP-DP) calculations [30]. Since the distributions of the points in the two dimensional plots of $\sqrt{LCL}$ (local community links) versus *CN* (common neighbors) for the complete network, and its first level modules show heterogeneities, the network and first level modules are heterogeneous in organization. The number of distinct patterns of the points in the plots indicate number of possible modules/sub-modules in the respective network and modules, and the distributions of the points reveal the organization in the respective network and modules. The extent and degree of spareness of the distributed points indicate the size and compactness (denser the points more in compact) of the network and modules respectively. Since the $\sqrt{LCL}$ versus *CN* plot of complete network has four distinct patterns, the network is composed of four distinct modules with different approximate compact sizes (extents of *CN*) 25, 60, 70 and 170 respectively (Fig 6 panels in upper most two rows). The network as well as the constituting modules have significantly large LCP-correlation values ([0.919 − 0.985] > 0.8) indicating the nodes are strongly connected. To understand energy distribution in the main network and the respective modules constructed from it i.e. modules at the first level of organization, we calculated Hamiltonian energy per node *H*_*s*_ using Eq (13), and found that *H*_*s*_ of all four modules are different indicating heterogeneity of energy distributions in the respective modules (Fig 6 first row right most panel). Further, *H*_*s*_ of two modules are found to be much larger than the other remaining two, and,

$$\begin{array}{c} \sum_{c=1}^{m^{\left[1\right]}}H_{N_{c}}^{\left[1\right]}<H_{N}^{\left[0\right]},\;H_{N_{c}}^{\left[1\right]}\neq H_{N_{c^{\prime }}}^{\left[1\right]},\;\forall c\neq c^{\prime } \end{array}$$ (14)

where, *m*^[1]^ is number of modules in the first level, and *N*_*c*_ is the size of cth module. Eq (14) is found to be true because of existence of isolated nodes in the first level modular organization.

**Fig 7:**
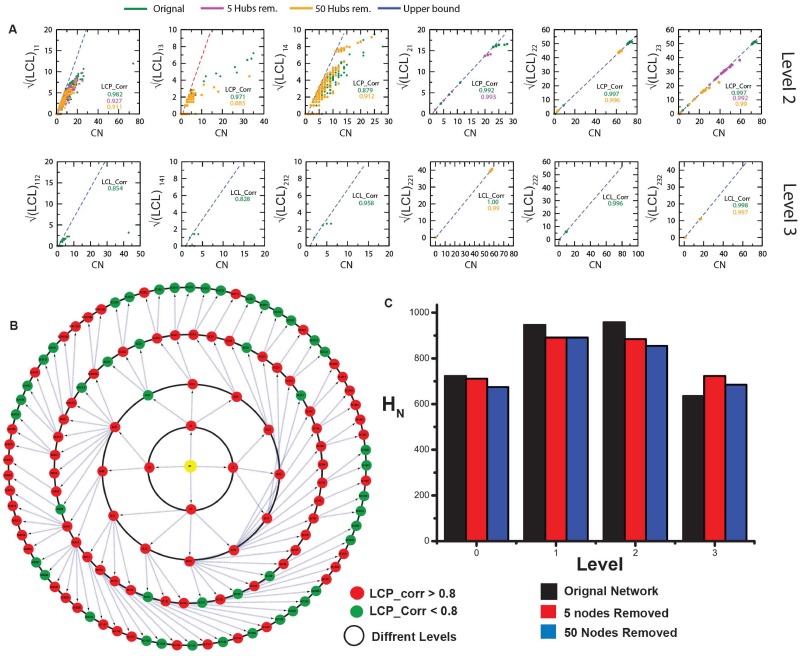
Compactness of breast cancer network: (a) LCP-correlation calculation as a function of CN for second and third level modules when zero, five and fifty leading hubs are removed. (b) Representation of modules/sub-modules based on the values of LCP-correlation values: modules with red color are for *LCP* − *corr* ≥ 0.8, and green color modules are for *LCP* − *corr* < 0.8.

We removed the first five leading hubs (which include three key regulators), and looked for changes in the network organization following the LCP-DP procedure. Even though there is a change in the compactness size of the four modules to 25, 52, 60 and 160 with respective LCP-correlation values [0.897 − 0.985] > 0.8 (Fig 6 panels in third and fourth rows), the network and its respective modules still strong compactness preserving most of the network properties (Fig 2). Similarly, we removed fifty leading hubs (including eleven fundamental key hubs) from the complete network, and the resulting network is still composed by four distinct modules with compact sizes 25, 35, 42 and 120 respectively with LCP-correlation values [0.866 − 0.975] > 0.8 (Fig 6 panels in the last two rows) still showing strong compactness and maintaining similar behaviors of the network properties (Fig 2). The calculated *H*_*N*_ in these networks (Fig 6 third and fifth rows right most panels) also show similar behavior as in Eq (14). We further continued this LCP-DP calculation for sub-modules at second and third levels (Fig 7 panels in the uppermost two rows) and still show strong compactness. This means that there could large number of key regulators which are in the comparatively low ranking category taking responsibilities of preserving the network properties and saving it from break down. Hierarchical networks generally have such properties and difficult to break it down. Now we calculate LCP-correlation of all the modules/sub-modules of the complete network distributed at various levels, and categorize them into two categories, 1. strong correlation, if the values of LCP-correlation values are larger than and equal to 0.8, and *weak correlation*, otherwise (LCP-correlation values<0.8) (Fig 7 lower panel). This result shows that the number of strongly correlated modules/sub-modules much larger than the number of weakly correlated modules/sub-modules at each level of organization in the network. This indicates that it is not easy to break down such networks.

### Origin of self-organization in the network

Breast cancer network follows hierarchical scale free nature (Fig 2) with strong modular organization (Fig 3). In this type of network, the topological parameters (*P*, *C*, *C*_*n*_, *C*_*B*_, *C*_*C*_, *C*_*E*_) which characterize network structure follows power laws (Fig 2 and Eqs (8) and (9)). The behaviors of these power laws can be represented by a single function Λ(*k*), which has the following scaling behavior [55, 56],

$$\begin{array}{c} \Lambda \left(\lambda k\right)=f\left(\lambda \right)\Lambda \left(k\right);\;f\left(\lambda \right)=\frac{\Lambda (\lambda k)}{\Lambda (k)}=\lambda^{D};\;D\to -\gamma ,-\alpha ,-\beta ,\epsilon ,\delta ,\mu \;\;as\;\;\Lambda \to P,C,C_{n},C_{B},C_{C},C_{E} \end{array}$$ (15)

This Eq (15) shows fractal nature of the topological properties of breast cancer network. The fractal behavior of the network is one important signature of self-organization [57] in this breast cancer network.

Further, nodes in breast cancer network are tighly bound (Fig 2) through various levels of network organization (Fig 3) and therefore cross-talk among them could be fast in the network. The popularities of leading hubs get changed on random basis at various level of organization (Fig 3), and the network shows disassortivity nature i.e. there is no signature of rich-club formation by these leading hubs. This means that there is no tendency to link up of leading hubs to regulate and rule the network, which is clear evidence of *absence of central control system*, which is one of the most important properties of self-organization [44].

The modules/sub-modules generated from the breast cancer network and distributed at various levels of organization are strongly compact as reflected from the high values of calculated LCP-correlations for all the modules/sub-modules (Figs 6 and 7). This strong compactness of the modules/sub-modules reveals that the constituting nodes in each module/sub-module are tightly bound, and are very sensitive in internal or/and external local and global fluctuations in it because of efficient information processing due to nodes’ strong inter-linked. This strongly contributes to the preservation of network properties against any internal or/and external changes or adapted to the favourable changes without breaking down the network, which is another one of the most important properties of self-organization [58, 59].

## Conclusion

Breast cancer network is found to follow hierarchical scale free network with strongly inter-linked modular/sub-modular features. The modules/sub-modules are organized hierarchically at different levels of organization preserving similar topological properties as the complete network has. Hence the modules/sub-modules at various levels of organization can be approximately seen as the replications of the complete network with some scale factor engineered by Eq (15)). At the same time the breast cancer network is, not only regulated by the highly inter-linked compact and heterogeneous modules/sub-modules but also by a large number of leading hubs of unpredictable change of their popularities with the level of organization. Out of these large number of leading hubs, only few are *key regulators* (FKR) which work at grassroots level for preserving the network properties and saving it from all possible local and global perturbations. These *FKR*s are in fact deeply rooted in the network, from complete network to motif level, and can be considered as the backbone of network in terms of signal processing, organizing/reorganizing among the nodes in the network, and modules/sub-modules at various levels of organization when the network is under attack, and adaptation to new fitted change. It is also found that some *FKR* have high popularities and some are not. However, the properties of the network does not merely change if there are large number of *FKR*s in it, and possibly the network may break down if all the *FKR*s are removed. In breast cancer network, we identified eleven *FKR*s (*EGFR, EP300, GRB2, BRCA1, KU70, RS3A, PHB, P53, CBP, RL21* and *RL13*) out of fifty leading hubs in it, which could be possible target genes.

In breast cancer network, *p53* gene is found to be a *FKR* which maintains low profile. However, the modules and sub-modules it passed through from complete network to motif level are mostly strongly compact in nature indicating p53 as fast information processor and key regulator which allows efficient cross-talk among the nodes in each module/sub-module it belongs. Even though its ranking is low i.e. eight among the identified *FKR*s in breast cancer network, it allows its modules/sub-modules at various level of organization to keep strong relative correlation with the complete network such that p53 provides strong relation from complete network to motif. The distributed energy per node in modules/sub-modules at various levels also is almost the same. At motive level, p53 also interact with two important genes, namely *BRCA2* and *BRCA3*. Hence p53 could be one of the most important *FKR*s in breast cancer network.

Breast cancer network maintains self-organization characterized by various properties, first, the topological properties of it follow fractal laws. Secondly, the removal of leading hubs does not cause network break down which is the evidence of absence of control mechanism. Third, the network and its constituent modules/sub-modules are strongly compact in nature, which could be a means of keeping network stabilization against any attack. Even though breast cancer network is self-organized one needs to have rigorous studies on *FKR*s for understanding this kind of disease and prevention.

## Supporting information

S1 Fig The structures of modules/sub-modules through which the first ten leading hubs passed through. The probability distribution of the seven fundamental key regulators as a function of level of organization.
(EPS)
